# Study of Brain Structure and Function in Chronic Mountain Sickness Based on fMRI

**DOI:** 10.3389/fneur.2021.763835

**Published:** 2022-01-07

**Authors:** Haihua Bao, Xin He, Fangfang Wang, Dongjie Kang

**Affiliations:** Department of Medical Imaging Center, Qinghai University Affiliated Hospital, Xining, China

**Keywords:** chronic mountain sickness, brain, hypoxia, voxel-based morphometry (VBM), amplitude of low frequency fluctuation (ALFF), functional MRI

## Abstract

**Objective:** Headache and memory impairment are the primary clinical symptoms of chronic mountain sickness (CMS). In this study, we used voxel-based morphometry (VBM) and the amplitude of the low-frequency fluctuation method (ALFF) based on blood oxygen level-dependent functional magnetic resonance imaging (BOLD-fMRI) to identify changes in the brain structure and function caused by CMS.

**Materials and Methods:** T1W anatomical images and a resting-state functional MRI (fMRI) of the whole brain were performed in 24 patients diagnosed with CMS and 25 normal controls matched for age, sex, years of education, and living altitude. MRI images were acquired, followed by VBM and ALFF data analyses.

**Results:** Compared with the control group, the CMS group had increased gray matter volume in the left cerebellum crus II area, left inferior temporal gyrus, right middle temporal gyrus, right insula, right caudate nucleus, and bilateral lentiform nucleus along with decreased gray matter volume in the left middle occipital gyrus and left middle temporal gyrus. White matter was decreased in the bilateral middle temporal gyrus and increased in the right Heschl's gyrus. Resting-state fMRI in patients with CMS showed increased spontaneous brain activity in the left supramarginal gyrus, left parahippocampal gyrus, and left middle temporal gyrus along with decreased spontaneous brain activity in the right cerebellum crus I area and right supplementary motor area.

**Conclusion:** Patients with CMS had differences in gray and white matter volume and abnormal spontaneous brain activity in multiple brain regions compared to the controls. This suggests that long-term chronic hypoxia may induce changes in brain structure and function, resulting in CMS.

## Introduction

Chronic mountain sickness (CMS) was initially described by Monge in 1928 and was later replicated by other groups in the early medical literature (1–6). It is a syndrome caused by an inability of individuals to adapt to high altitudes (altitude >2,500 m) and is typically manifested as hyperhemoglobinemia and hypoproteinemia (7). Common symptoms include headache, dizziness, insomnia, fatigue, tinnitus, attention deficit, amnesia, and muscle and joint pain. Some patients may also have cerebral edema, eventually leading to encephalopathy (8). Hypoxia is an important cause of CMS due to low oxygen levels in high-altitude areas. In recent years, with an increase in the plateau population, CMS has received greater attention (9, 10). Most published literature has focused on the epidemiology, pathophysiology, and genetics (11–16) of CMS. In contrast, there is little research on imaging, except for a few studies, such as the one by Bao et al., which evaluated brain damage in patients with CMS. The authors found that multiple structural changes occur in the brains of patients with CMS and are correlated to the severity of the disease and the impairment of cognitive functions (17). Wei et al. suggested that the differences in cortical morphometry in the brains of native Tibetans may reflect adaptations related to high altitudes (18). Furthermore, imaging studies of plateau residents have been limited to normal controls. Structural MRI studies of adolescents migrating from sea level to a high altitude environment demonstrated the following changes: (a) decreased gray matter volume in the right postcentral gyrus, right superior frontal gyrus, bilateral anterior insula, right anterior cingulate cortex, bilateral pre-frontal cortex, left precentral cortex, and right lingual cortex, and (b) increased gray matter volume in the right middle frontal gyrus, right parahippocampal gyrus, right inferior and middle temporal gyri, bilateral inferior ventral pons, and right cerebellum crus I (19, 20). A study on adolescents migrating from a high altitude to sea level environment demonstrated the following changes: (a) increased gray matter volume in the left insula, left inferior parietal gyrus, and right superior parietal gyrus, (b) decreased gray matter volume in the left precentral cortex and multiple sites in the cerebellar cortex (left lobule VIII, bilateral lobule VI, and crus I/II), and (c) decreased white matter volume in the right superior frontal gyrus (21). Studies using functional MRIs (fMRIs) on long-term high-altitude residents have indicated that subjects' brains undergo functional changes (22, 23); the left pyramis, and left and superior temporal gyrus were more activated, and the left middle occipital gyrus was less activated in the high-altitude group than in the control group (22). High-altitude residents also showed a decreased activation in the inferior and middle frontal gyrus, the middle occipital and lingual gyrus, the pyramis of the vermis, and the thalamus (23). Our research aims to study structural and functional changes simultaneously, using voxel-based morphometry (VBM) and the amplitude of low-frequency fluctuation (ALFF) methods, from resting-state fMRI in patients with CMS. The VBM provides a quantitative and comprehensive assessment of anatomical changes in the brain (24). This technique has been widely used in the study of changes in the brain morphology caused by various diseases (25–28). Currently, the methods for analyzing resting-state blood oxygen level-dependent functional magnetic resonance imaging (BOLD-fMRI) data are regional homogeneity (ReHo), ALFF, and independent component analysis (ICA). The ALFF method has been widely used in clinical settings (29–31) to deduce information about the amplitude of spontaneous activity of regional neurons (32, 33). To the best of our knowledge, no studies have used both structural MRI volumetrics and resting-state fMRI to study CMS.

## Materials and Methods

### Subjects

After being assessed at our institution between May 2017 and May 2020, 25 patients diagnosed with CMS were selected for this study. One patient was excluded because of excessive head motion during the MRI. The currently used international diagnostic criteria for CMS are the Qinghai criteria (34), which include indicators for hemoglobin and various clinical symptoms. None of the patients received any treatment for CMS before MRI examination. Control subjects (*n* = 25) with comparable age, educational background, and high-altitude exposure were recruited. Physical examination showed no positive signs of CMS and a normal hemogram and blood pressure in the control subjects. All subjects included in the study were right-handed and had lived in a plateau at altitudes above 3,000 m for at least 5 years. The subjects were native highlanders from the Qinghai Province and had no documented drug abuse, high blood pressure, diabetes, neurological disorders, or history of head injuries such as loss of consciousness and mental illness. Men are reportedly more prone to CMS than women (35–37). To avoid sex-related differences, we recruited only men in our study. The experimental protocol was approved by the ethics committee of our institution. All subjects provided written informed consent before participating in the study.

### Equipment and Technical Indicators

The MRI scans were conducted using a Philips Achieva 3.0 TX scanner with an eight-channel coil. Anatomical MRI scans were performed on all subjects, including fluid attenuated inversion recovery (FLAIR), T2-weghted imaging (T2WI), and diffusion-weighted imaging (DWI). Further scans were performed on subjects who had no abnormalities on baseline images that could interfere with quantitative analysis and interpretation of the resting-state fMRI. A turbo field echo (TFE) sequence was used to obtain three-dimensional (3D)-T1 images with the following parameters: TR = 7.5 ms, TE = 3.5 ms, excitation angle: 7°, matrix: 256 × 256, transverse scanning, slice thickness: 2 mm, 176 images. Using the echo-planar imaging (EPI) sequence (TR/TE = 2,500/30 ms, FOV = 224 mm X 224 mm, flip angle = 90), axial slices of 3.5 mm thickness covering the whole brain were applied to each subject to acquire the BOLD fMRI data (scan time: 385 s).

### Data Processing

#### VBM Method

Data were processed using the VBM8 toolbox implemented in SPM8 (Wellcome Department of Imaging Neuroscience, University College London, London, UK). The analysis was performed using MATLAB (MathWorks, Natick, MA, USA). The following steps were included: (1) Each scan was visually examined for scanning artifacts and anatomic abnormalities, (2) The plane of the image was adjusted to the anterior commissure (AC) and posterior commissure (PC) lines on the transverse plane (3). Each reoriented image was segmented into gray matter, white matter, and cerebrospinal fluid. Diffeomorphic anatomical registration through exponentiated lie algebra (DARTEL) was used to achieve a high dimension of registration and standardization, and (4) normalized images were transformed into Montreal Neurological Institute (MNI) space. Gray matter images were then smoothed using a Gaussian kernel of 8 mm full width at half maximum (FWHM).

#### ALFF Method

The fMRI data were preprocessed using a data processing assistant for resting-state fMRI (DPARSF). The first 10 time points were discarded to stabilize the signals. One patient was excluded due to head motion of >1.5 mm and 1.5 degrees. After processing, the data for each individual were spatially normalized to the MNI space. Images were resampled to 3 mm and smoothed with a kernel of 8 mm FWHM. The REST package (REST, http://resting-fmri. sourceforge.net) was used to calculate the ALFF using a voxel-based approach. The power spectrum was obtained by square-rooted FFT and averaged across 0.01–0.08 Hz at each voxel. The averaged square root was used as the ALFF. To reduce the global effects of variability across the participants, the ALFF of each voxel was divided by the global mean ALFF value obtained previously within the whole-brain mask.

### Statistical Analysis

#### VBM Analysis

A two-sample *t*-test was conducted between the patients and control groups. A statistical parametric map was generated at |t| > 2.0 1, *P* < 0.05, cluster > 50.

#### ALFF Analysis

Two-sample *t*-test was conducted on the groups. The statistical parametric map was generated at *P* < 0.05, cluster > 100 (uncorrected).

## Results

### Demographic and Physiological Information

The demographic and physiological data of the CMS and control groups, including age, body weight, altitude, education, CMS score, and hematological parameters (HGB, RBC), are shown in Table 1.

**Table 1:** Demographic and physiological data.

	**CMS group**	**Control group**	***P* value**
Number of subjects	24	25	
Age (years)	41.46 ± 1.77	46.08 ± 1.70	0.083
Body Weight (kg)	75.02 ± 2.65	73.70 ± 2.59	0.467
Altitude (m)	3450 ± 585	3556 ± 432	0.544
Education (years)	10.50 ± 5.93	13.55 ± 4.66	0.1 8
CMS score	12.71 ± 3.07	3.82 ± 1.17	0.07
**Hematological measurements**			
HGB (g/L)	215.13 ± 6.47	178.32 ± 6.09	<0.001
RBC (x10^12^)	6.91 ± 0.19	5.76 ± 0.19	<0.001

*Data are mean ± SD.Chronic mountain sickness (CMS) score based on Qinghai criteria (reference 32)*.

### Detailed Information of CMS Patients

Detailed information of the patients with CMS, including the altitude where they lived (in meters), hematological parameters (HGB, RBC), CMS score, and duration of residence at high altitude, is shown in Table 2.

**Table 2:** Detailed information of the patients with chronic mountain sickness (CMS).

**Names of**	**Altitude**	**HGB**	**REB**	**CMS score**	**Residence time**
**Patients**	**Meter**	**g/L**	**10^**12**^/L**	**degree**	**year**
Subject 1	3,500	223	7.29	Mild	Generational residence
Subject 2	3,700	227	8.42	Moderate	6
Subject 3	2,500	220	7.28	Mild	Generational residence
Subject 4	4,100	255	7.47	Mild	Generational residence
Subject 5	3,800	257	7.76	Moderate	13
Subject 6	3,700	235	7.18	Mild	13
Subject 7	3,700	207	7.16	Mild	10
Subject 8	3,450	220	7.00	Mild	Generational residence
Subject 9	4,600	222	7.50	Mild	8
Subject 10	3,550	240	7.10	Moderate	Generational residence
Subject 11	2,500	239	8.57	Severe	13
Subject 12	3,000	232	7.83	Moderate	Generational residence
Subject 13	3,300	264	8.39	Moderate	7
Subject 14	4,200	211	6.35	Mild	6
Subject 15	3,800	224	6.27	Mild	Generational residence
Subject 16	4,000	218	6.90	Mild	Generational residence
Subject 17	4,000	242	7.54	Mild	Generational residence
Subject 18	4,000	246	8.10	Moderate	Generational residence
Subject 19	3,500	215	6.80	Mild	generational residence
Subject 20	2,600	230	7.13	Moderate	Generational residence
Subject 21	3,200	235	7.40	Moderate	Generational residence
Subject 22	2,600	228	7.21	Moderate	Generational residence
Subject 23	2,800	230	7.33	Moderate	Generational residence
Subject 24	3,000	243	7.60	Severe	Generational residence

*Generational residence = lifelong resident in the high altitude region*.

### VBM Result

**Figure 1:**
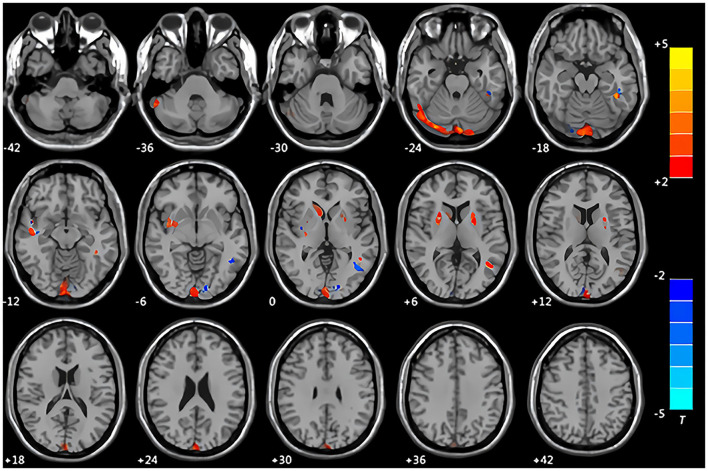
Maps of changed brain regions of gray matter in patients with chronic mountain sickness (CMS) compared with the control group. Areas in red are regions where gray matter volume was significantly increased: left cerebellum crus II area, left inferior temporal gyrus, right insula, right caudate nucleus, bilateral lentiform nucleus. Areas in blue are regions where gray matter volume was significantly decreased: left middle occipital gyrus, left middle temporal gyrus.

**Figure 2:**
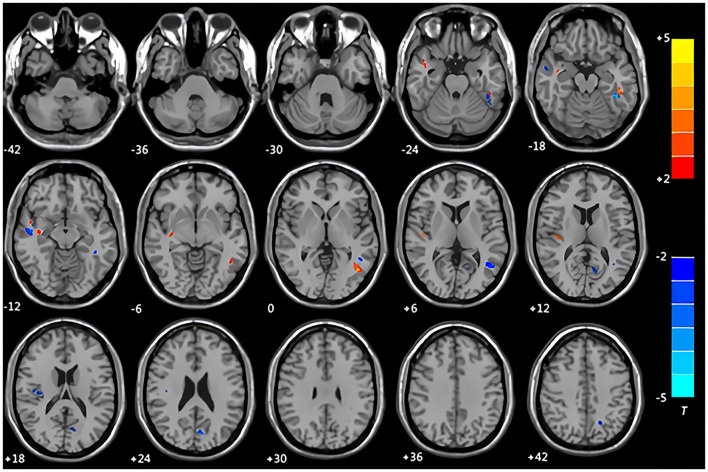
Maps of changed brain regions of white matter in patients with CMS compared with the control group. Areas in blue are regions where white matter volume was significantly decreased in the bilateral middle temporal gyrus. Areas in red are regions where white matter volume was significantly increased in the right Heschl's gyrus.

Compared with the control group, the patients with CMS showed an increase in gray matter volume in the right cerebellum crus II area, left inferior temporal gyrus, right middle temporal gyrus, right insula, right caudate nucleus, and bilateral lentiform nucleus. Volume was decreased in the left middle occipital gyrus and left middle temporal gyrus (Table 3, Figure 1). White matter was decreased in the bilateral middle temporal gyrus and increased in the right Heschl's gyrus (Table 4, Figure 2).

**Table 3:** Regional information of altered gray matter volume.

**Regions**	**Voxels**	**MNI coordinates**			**t-score (peak)**
		**X**	**Y**	**Z**	
Cerebellum crus 2 area_R	506	30	−84	−21	5.3735
Inferior temporal gyrus_L	61	−42	−42	−16	5.393
Insula_R	82	40	−4	−4	3.8444
Middle temporal gyrus_R	52	48	−14	−12	3.7036
Caudate nucleu_R	51	12	14	0	2.7356
Putamen_L	58	−22	14	6	3.1452
Putamen_R	53	26	0	6	3.3476
Middle Occipital gyrus _L	58	−14	−90	−4	−3.435
Middle temporal gyrus_L	115	−42	−62	2	−4.2172

**Table 4:** Regional information of altered white matter volume.

**Regions**	**Voxels**	**MNI coordinates**			**t-score (peak)**
		**X**	**Y**	**Z**	
Middle temporal gyrus_R	134	48	−12	−12	−3.452
Middle temporal gyrus _L	125	−48	−60	10	−4.3143
Heschl_R	55	42	−20	10	3.0061

### ALFF Result

**Figure 3:**
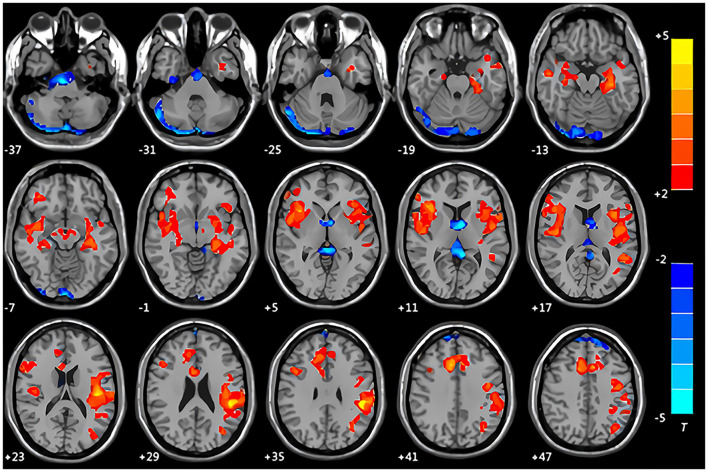
Maps of the amplitude of the low-frequency fluctuation (ALFF) changes in patients with CMS compared with the control group. Areas in red are regions where ALFF value was significantly increased: left supramarginal gyrus, left parahippocampal gyrus, left middle temporal gyrus. Areas in blue are regions where ALFF value was significantly decreased: right cerebellum crus I area, right supplementary motor area.

Compared with the control group, the patients with CMS in the resting state showed increased spontaneous brain activity in the left supramarginal gyrus, left parahippocampal gyrus, and left middle temporal gyrus along with decreased spontaneous brain activity in the right cerebellum crus I area and right supplementary motor area (Table 5, Figure 3).

**Table 5:** Regional information of changed amplitude of the low-frequency fluctuation (ALFF).

**Regions**	**Voxels**	**MNI coordinates**			**t-score (peak)**
		**X**	**Y**	**Z**	
Supramarginal gyrus_L	2,881	−48	−33	30	5.2726
Parahippocampa gyrus_L	164	−30	−24	−18	3.9926
Middle temporal gyrus_L	110	−48	−45	10	3.0787
Supplementary motor area_R	665	3	6	75	−3.6227
cerebellum crus 1 area _R	834	54	−66	−33	−3.0982

## Discussion

### Physiological and Biochemical Effects of Hypoxia on Human Brain Tissue

The human cerebral cortex has been shown to have features of neuroplasticity with a structure and function that can be modified and adapted to different stimuli (38). Certain physical and environmental states can lead to alterations in respiratory and circulatory function, hemoglobin concentration, and arterial oxygen saturation with subsequent changes in cerebral blood flow, thereby leading to cumulative changes in brain structure (39). The brain responds to hypoxia by regulating the changes in the cardiovascular and respiratory systems; therefore, the physiological response to hypoxia may lead to structural changes in various brain regions (20). The microstructure of gray matter is a complex mixture of nerve cells, fibers, neuroglial cells, and vessels. An increase in gray matter volume may be associated with neural cell hyperplasia, increased synapses, or changes in the cerebral vasculature (40). Under hypoxic conditions, however, the decrease in gray matter volume may be associated with the byproducts of metabolism and increased glutamate release from nerve cells (41). The new cortex can regenerate, which can be induced by hypoxia and cerebral ischemia, stimulating the proliferation of microglia and macrophages (42, 43).

### The Implications of Changes in Gray Matter

In this study, we investigated the brain structure and functional changes in patients with CMS using VBM and resting-state fMRI. Our results demonstrate that the patients with CMS had increased gray matter in the right cerebellum crus II area, left inferior temporal gyrus, right insula, right caudate nucleus, and bilateral lentiform nucleus along with decreased volume in the left middle occipital gyrus and left middle temporal gyrus. These findings were similar to the results of a study by Zhang et al., which found increased gray matter in the right cerebellum crus II area and right middle temporal gyrus. The insular cortex has been shown to be associated with the control of cardiovascular disease, while the frontal island plays an important role in dyspnea, which is a symptom demonstrated by most patients with CMS (44–47). Several studies have shown that the frontal island is necessary to maintain homeostasis in high-altitude environments. There is a strong correlation between the right frontal insula gray matter and aerobic capacity and significant activation of the insula with dyspnea on fMRI (48–50). In our study, the CMS group showed increased gray matter volume in the insula, which could explain the dyspnea symptoms in patients with CMS. Other brain functional studies on hypoxic patients suffering from a chronic obstructive pulmonary disease (COPD) or obstructive sleep apnea (OSA) also revealed changes in gray matter volume, similar to the findings of our study (51–53).

### The Implications of Changes in White Matter

Our results demonstrated decreased white matter volume in the bilateral middle temporal gyrus. There was a decrease in gray matter volume in the left middle temporal gyrus. These findings suggest that the middle temporal gyrus may be more sensitive to long-term hypoxia, as seen in patients with CMS. In this study, changes in gray matter were greater than those in white matter, indicating that gray matter was more sensitive to hypoxia in patients with CMS than white matter (38).

### The Implications of Changes in ALFF

The resting-state analysis of patients with CMS showed increased spontaneous brain activity in the left supramarginal gyrus, right central sulcus cover, left parahippocampal gyrus, and left middle temporal gyrus, while the right cerebellar amygdala and right supplementary motor area showed decreased spontaneous brain activity. Hypoxia has been shown to increase nerve cell activity and volume in the hippocampus, which suppresses the normal physiological response to hyperventilation, thereby aggravating the hypoxic state (47, 54–57). One study showed that adolescents migrating from sea level to high-altitude environments had increased gray matter in the hippocampus (19). Our results, using resting-state fMRI, showed increased spontaneous brain activity in the hippocampus of patients with CMS, inducing memory decline (19). Recent research has shown that the cerebellum is also involved in cognition, language, and emotion regulation (58, 59). Our study demonstrated increased ALFF activity and altered gray matter volume in the right cerebellum crus I area, which may be associated with changes in cognitive function, emotional disorder, and irritability in patients with CMS. Future studies with neuropsychological testing in our patient cohorts may be helpful to correlate the posterior fossa findings with altered resting-state fMRI and volumetrics compared to controls in terms of the neuropsychological changes described above.

Our study has multiple limitations. First, we investigated a small sample size of patients with CMS, and this may have skewed the results. Second, we did not perform neuropsychological tests in our patient cohorts to correlate our imaging findings with clinical manifestations. Third, white matter tract integrity may also be affected by hypoxia in patients with CMS. Future studies can also study white matter integrity using DTI tractography in patients with CMS.

In conclusion, the purpose of this study was to investigate changes in the brain structure using volumetrics and brain function using resting-state fMRI in patients with CMS due to long-term hypoxia. The results demonstrated altered gray matter volume, white matter volume, and brain function in patients with CMS compared to the controls, which were most likely related to long-term hypoxia. Therefore, this study shows the effects of long-term hypoxia on brain tissue from the perspective of imaging using volumetrics and resting-state fMRI.

## Data Availability Statement

The original contributions presented in the study are included in the article/supplementary material, further inquiries can be directed to the corresponding author.

## Ethics Statement

The studies involving human participants were reviewed and approved by Ethics Committee of Qinghai University Affiliated Hospital. The patients/participants provided their written informed consent to participate in this study.

## Author Contributions

HB, FW, and XH: conception and design. HB: administrative support and final approval of manuscript. FW and XH: provision of study materials or patients, collection and assembly of data, data analysis, and interpretation. HB and XH: funding acquisition. XH and DK: software and validation. XH and HB: supervision. XH: visualization. XH and FW: manuscript writing. All authors contributed to the article and approved the submitted version.

## Funding

This study was funded by the Science and Technology Project of Qinghai Province (No. 2017-SF-158).

## Conflict of Interest

The authors declare that the research was conducted in the absence of any commercial or financial relationships that could be construed as a potential conflict of interest.

## Publisher's Note

All claims expressed in this article are solely those of the authors and do not necessarily represent those of their affiliated organizations, or those of the publisher, the editors and the reviewers. Any product that may be evaluated in this article, or claim that may be made by its manufacturer, is not guaranteed or endorsed by the publisher.
